# Is monkeypox a threat to another pandemic?

**DOI:** 10.3389/fmicb.2022.983076

**Published:** 2022-08-31

**Authors:** Kingshuk Panda, Anupam Mukherjee

**Affiliations:** ^1^Indian Institute of Technology, New Delhi, India; ^2^Division of Virology, ICMR-National AIDS Research Institute, Pune, MH, India

**Keywords:** monkeypox, outbreak, pandemic, epidemic, infectious disease

The world has witnessed some of the deadliest viral epidemics and pandemics during this 21^st^ century; HIV, Swine Flu or pH1N1, MERS-CoV, Ebola, Zika, Chikungunya, Dengue, and most recently SARS-CoV-2 or COVID-19 are recorded as few of the many with far-reaching consequences. By Aug 12, 2022, the SARS-CoV-2 virus has been responsible for more than 585 million COVID-19 cases globally, resulting in 6.4 million deaths (WHO, 2022c). In reality, the wounds that the COVID-19 pandemic has inflicted on us are very deep and seem to be long-lasting. Amid this ongoing pandemic situation, the increasing cases of monkeypox incidence are becoming a global threat. The virus called monkeypox causes a rare disease in monkeys and humans, specifically in the regions of central and western African countries. In 1958, the virus was first isolated in a laboratory when scientists found pox-like outbreaks in monkeys that were kept for research purposes (Von Magnus et al., 1959). The major animal reservoir or monkeypox is still not discovered, although few studies suggested Gambian pouched rats and rope squirrels are the most suspected reservoir (CDC, 2003; Brown and Leggat, 2016). Previously, in 2003, an outbreak was recorded in the US regions, where most of the cases were reported in both humans and pet prairie dogs (Ligon, 2004). Some sporadic outbreak of monkeypox have been reported in 2018, 2019, and 2021 (Kraemer et al., 2022). Although the number was minimal, most cases were reported in the same family. As per the latest report on Aug 5, 2022, a total of 28,220 confirmed cases have been reported to the World Health Organization (WHO) from 88 countries, out of that 81 countries were not under the monkeypox virus endemic zone earlier. As of now, there are 2859 active cases in the United Kingdom, 7509 cases in Unites states of America, 4942 cases in Spain, and 2887 cases in Germany.

There are two possible routes for monkeypox transmission. These are animal-human transmission and human-human transmission. The animal-to-human transmission is known as zoonotic transmission, which occurs *via* direct contact or through food, water or the environment (Bunge et al., 2022). A few nosocomial transmissions have also been reported in several regions of Africa. Though the monkeypox virus can enter through large respiratory droplets, close or direct contact with skin lesions, and possibly through contaminated fomites, most of the cases of 2022 outbreaks have been identified in the primary care and sexual health settings, mostly among the men who have sex with men groups (MSM) (Kupferschmidt, 2022; WHO, 2022b). The virus generally replicates at the inoculation site and then spreads to the local lymph nodes. The incubation period generally took around 6 to 13 days with a maximum upper limit of 21 days. There is usually a 0-5 day invasion period, accompanied by fever and lymphadenopathy before lessons begin (WHO, 2022a). Persons may experience flu-like symptoms during the prodrome phase, such as fever, chills, malaise, headaches, backaches, sore throats, shortness of breath, and swollen lymph nodes. The enlarged lymph nodes are the unique clinical clue of the monkeypox virus, which distinguish it from human smallpox infection caused by the variola virus (Cann et al., 2013). The rash is generally well circumscribed, vesicular, or pustular that are deep-seated, firm or hard. Over time, the lesions may umbilicate or become confluent, forming scabs. Although, recent cases have suddenly started without symptoms like fever, genital lesions, or other prodromal symptoms (Minhaj et al., 2022). As the clinical signs and symptoms of monkeypox are similar to smallpox, chickenpox, measles, coxsackievirus and bacterial infection in the early period, hence it is very important to use a differential diagnosis of monkeypox in the early period. Serological methods are not recommended for monkeypox diagnosis due to the cross-reactivity of other orthopoxviruses, therefor Polymerase Chain Reaction (PCR) is the most recommended method for genome-level identification with higher accuracy and sensitivity (Saxena et al., 2022).

Currently, no licensed treatment is available for monkeypox, although two oral drugs, brincidofovir, and tecovirimat, have been approved to treat smallpox and show antiviral efficacy against monkeypox (Adler et al., 2022). A study published in Lancet reported that one patient treated with tecovirimat, with a dosage of 200 mg twice daily for 2 2 weeks orally, showed a short duration of viral shedding and illness upon comparison with other patients (Adler et al., 2022). In addition, the vaccine named MVA-BN, also known as JYNNEOS^TM^ in the US, has been licensed in the United States to prevent the cases of monkeypox or make it less severe. In Canada, the vaccine is called IMVAMUNE®, while in the European Union it is marketed under the trade name IMVANEX® to reduce monkeypox severity and prevent future infections. Recently, the Center for Disease Control and Prevention (CDC) published a datasheet for monkeypox treatment, where they reported that the smallpox vaccine, cidofovir, ST-246, and vaccinia immune globulin can be used to control the monkeypox outbreak but no supporting data is attached to their claim (CDC, 2022). Therefore, the development of proper vaccines or antivirals against the monkeypox infection is the need of the hour that leads toward detailed research on the infection biology of the virus. Although a few papers have already reported monkeypox's infection biology, there is an urgent need to understand the virus life cycle starting from the role of different cellular organelles in viral entry into the cell, each step of its replication machinery, detailed interactions with the host cells, trafficking, and finally the egress of the mature viruses (Satheshkumar and Moss, 2012; Sivan et al., 2016; Realegeno et al., 2020). Equally, more research on the epidemiological forecasts of the virus, transmission dynamics and therapeutics prospects are required to understand the proper model of disease management for monkeypox infection. Recently, In 2021, a research group designed a mathematical model to understand the transmission dynamics of the monkeypox virus (Peter et al., 2021). Still, there are many more stones to be turned to understand the proper way for the management of monkeypox.

The world has already faced a significant outbreak since the last quarter of 2019. Therefore, there is a panic about whether monkeypox could cause such a COVID-19-like pandemic or not. The SARS-CoV-2 virus spreads through tiny airborne droplets called aerosols, whereas monkeypox is mainly spread from close contact with a body fluid such as saliva and coughing. Although emerging literature points toward the presence of monkeypox DNA in the short-range aerosols, the efficiency of this transmission route is still under subjected to further research and validation. Besides everything, any viral disease's primary concern is its new behavior. If we recall, in the initial pandemic situation of COVID-19, there were a few mutations identified. Nevertheless, later on, scientists cataloged more than 12,000 mutations in the SARS-CoV-2 genome. The monkeypox virus genome consists of linear double-stranded DNA, size of approximate ~197 kb. The genome of this virus is six times as large and complex to analyze as compared to the genome of the SARS-CoV-2 coronavirus (Kozlov, 2022). As the genome of monkeypox is DNA; therefore, the DNA polymerase contains proofreading skills, which makes DNA viruses prone to being less mutagenic; hence, we hypothesize that the monkeypox infection is less likely to create another pandemic situation like COVID-19. However, there are evidences of certain mutations found in the monkeypox DNA sample (Weaver and Isaacs, 2008; Zhang et al., 2022). Previously, a study demonstrated that COP-C3L is a major gene responsible for the difference in virulence among different monkeypox strains, and predicted that mutation in this gene could raise a significant pathogenic strain of monkeypox (Weaver and Isaacs, 2008). A latest analysis indicated the occurrence of single nucleotide mutations and frame-shift mutations in the samples collected from this outbreak (Zhang et al., 2022). Therefore, whether monkeypox could lead to another pandemic at this time point is still a debatable subject.

Few hypotheses have already risen regarding this sudden increase in monkeypox cases. Few studies say that the current sequences retrieved are mostly similar to those from a smattering of monkeypox cases that arose outside Africa in 2018-2019 and are linked with the traveling history. Another hypothesis says that there must be a possibility that the virus was circulating outside Africa in humans and animals but remains undetected (Kozlov, 2022). Whereas, a third hypothesis says that the virus may be coincidently exposed to the community by sexual networks as the recent unexpected cases in MSMs have increased (Mohapatra et al., 2022).

After decades of quiescence, the human monkeypox disease has become a clinically serious infection (Costello et al., 2022). Since the disease was first reported, no extensive studies have been conducted on the exploitation of the host cells by monkeypox infection. Although, few reports have been published to identify the mechanism of host susceptibility to monkeypox infection upon exposure, specifically in mouse models. The common inbred strains of mice, including BALB/c and C57BL/c, are remarkably resistant to monkeypox virus infection, however, CAST/EiJ shows greater sensitivity and excellent morbidity and mortality due to their inadequate immune response upon monkeypox infection (Americo et al., 2010; Earl et al., 2012, 2015). The classical inbred mice make a more active interferon γ- response, which makes them less susceptible. It has also been proven that the cytokine IL-15 and the number of NK cells play a critical role in combating monkeypox infection (Earl et al., 2020). At present, there are some crucial gaps in understanding the host-cell biology, pathophysiology and epidemiology of the Monkeypox virus.

Besides all of these, a few standard measures should be taken by the public to prevent infection with monkeypox. Moreover, recommendations from WHO is necessary at this point to increase awareness among the common people. Although, a few common principles are the thumb rule to prevent any such viral diseases; Firstly, separate an infected person from a healthy person; Secondly, utilize appropriate protective equipment and good hand hygiene to protect household members when dealing with the infected individuals or serving as caregiver at home; Thirdly, for disinfection of surface, use an EPA-registered disinfectant; Fourthly, patients should avoid contact with pets and animals while infected, as animals are a potent reservoir of monkeypox; Finally, monkeypox symptoms, including unexplained lesions, should be evaluated by a dermatologist and venereologists, and close contact with others, including sexual or intimate partner, should be avoided until the condition is evaluated (Khanna et al., 2022; Minhaj et al., 2022).

Furthermore, we must avoid stigmatizing any infected individual for the source of their infection. An important reason for this outbreak is the inveterate neglect of diseases primarily affecting the poorest populations and the widespread disregard for communities affected by these diseases (Nakoune and Olliaro, 2022). The increasing evidence requires further research on the virus-cell interactions and investigation to understand the disease dynamics. It is crucial to provide proper interventions that prove effective in monkeypox endemic low-income countries, and not simply stockpile them for potential use in high-income countries. The world has already faced a global pandemic; therefore, it should be essential to be alert and ready to respond rapidly.

## Author contributions

All authors listed have made a substantial, direct, and intellectual contribution to the work and approved it for publication.

## Funding

This review work has been supported by the Indian Council of Medical Research and ICMR-National AIDS Research Institute, Pune.

## Conflict of interest

The authors declare that the research was conducted in the absence of any commercial or financial relationships that could be construed as a potential conflict of interest.

## Publisher's note

All claims expressed in this article are solely those of the authors and do not necessarily represent those of their affiliated organizations, or those of the publisher, the editors and the reviewers. Any product that may be evaluated in this article, or claim that may be made by its manufacturer, is not guaranteed or endorsed by the publisher.
